# A Novel Method in Identifying Pyroptosis and Apoptosis Based on the Double Resonator Piezoelectric Cytometry Technology

**DOI:** 10.3390/bios13030356

**Published:** 2023-03-07

**Authors:** Wenwei Li, Jing Li, Yanyang Wu, Tiean Zhou

**Affiliations:** 1 College of Bioscience and Biotechnology, Hunan Agricultural University, Changsha 410128, China; 2 Hunan Provincial Engineering Technology Research Center for Cell Mechanics and Function Analysis, Changsha 410128, China; 3 College of Food Science and Technology, Hunan Agricultural University, Changsha 410128, China

**Keywords:** double resonator piezoelectric cytometry, cell pyroptosis, cell apoptosis, HeLa cell, cell-exerted stress, cell viscoelasticity

## Abstract

In this study, a double resonator piezoelectric cytometry (DRPC) technology based on quartz crystal microbalance (QCM) was first employed to identify HeLa cell pyroptosis and apoptosis by monitoring cells’ mechanical properties in a real-time and non-invasive manner. AT and BT cut quartz crystals with the same frequency and surface conditions were used concurrently to quantify the cells-exerted surface stress (Δ*S*). It is the first time that cells-exerted surface stress (Δ*S*) and cell viscoelasticity have been monitored simultaneously during pyroptosis and apoptosis. The results showed that HeLa pyroptotic cells exerted a tensile stress on quartz crystal along with an increase in the elastic modulus (*G*′), viscous modulus (*G*″), and a decrease of the loss tangent (*G*″/*G*′), whereas apoptotic cells exerted increasing compressive stress on quartz crystal along with a decrease in *G*′, *G*″ and an increase in *G*″/*G*′. Furthermore, engineered GSDMD^−/−^-DEVD- HeLa cells were used to investigate drug-induced disturbance and testify the mechanical responses during the processes of pyroptosis and non-pyroptosis. These findings demonstrated that the DRPC technology can serve as a precise cytomechanical sensor capable of identifying pyroptosis and apoptosis, providing a novel method in cell death detection and paving the road for pyroptosis and apoptosis related drug evaluation and screening.

## 1. Introduction

Apoptosis is genetically controlled programmed cell death (PCD), which is responsible for the removal of damaged cells from the organism without triggering inflammation. It is related to the onset and progression of cardiovascular diseases, cancers, and other disorders [1,2,3]. In cancer intervention, tumor cells’ resistance to apoptosis is the primary cause of poor treatment response [4], whereas pyroptosis, which is characterized by an obvious inflammatory response, appears to be an alternative that bypasses apoptosis and activates tumor-specific immune responses, resulting in remarkable tumor regression [5], with less than 15% pyroptosis required for tumor clearance [6]. As a pro-inflammatory necrosis, pyroptosis is associated not only with cardiovascular diseases and cancers, but also with numerous infectious diseases (including COVID-19 and HIV/AIDS) [7,8,9,10]. The identification of pyroptosis and apoptosis is of utmost relevance since pyroptosis and apoptosis both play a crucial role in disease regulation, but induce diverse function effects and biological responses that result in disparate disease progression. Similarities between pyroptosis and apoptosis are restricted to chromatin condensation and DNA fragmentation, but distinctions include the activation mechanism: biochemical production to morphological phenotype. Morphologically, apoptosis is characterized by cell shrinkage, cellular sequestration, and membrane blebbing but with an intact membrane whose contents are dismantled and cleared in apoptotic blebs. In contrast, pyroptosis is distinguished by cell swelling, volume expansion, loss of membrane integrity, and cell lysis. Observation with high-tech spectroscopy, such as electron microscopy, laser scanning confocal microscopy, and others [11], is the most intuitive way for identifying pyroptosis and apoptosis based on morphological differences. Although these methods are straightforward and convenient, the identification standard is subjective and observational range is constrained. Consequently, the prevalent identification methods/technologies are mainly based on molecular distinctions. Primarily, Western blot assays, ELISAs, and immunofluorescence are used to detect protein expression, whereas DNA gel electrophoresis, RT-qPCR, and TUNEL are used to assess genomic expression. These methods can perform quantitative or semi-quantitative assays; however, they are not sensitive enough to avoid false-positive results. The flow cytometry assay is more sensitive than previous technologies, but it is costly, complicated to operate, and involves phototoxicity that cannot be eliminated during the detection process. Identifying pyroptosis and apoptosis using mechanical technology could therefore be considered an option. To date, cell mechanics in pyroptosis and apoptosis have been captured by dedicated studies utilizing various mechanical technologies [12,13]; these approaches are sensitive, but most of them are based on single-cell measurement, which falls short of quantitative analysis requirements (Supplementary Table S1). Notably, the technologies are mostly dependent on end-point detection, and there is a lack of real-time and non-invasive ways for identifying the number of cells undergoing pyroptosis and apoptosis. 

**Scheme 1:**
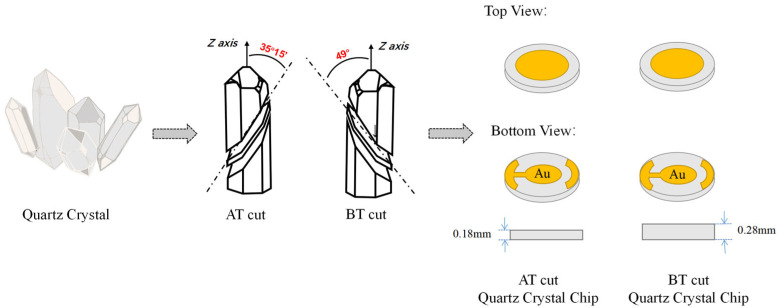
Schematic illustrations of AT and BT cut crystals. AT cut quartz crystal is cut at an angle of 35°15′ relative to the Z axis, whereas BT cut quartz crystal is cut at an angle of −49° relative to the Z axis. The crystal blanks are then cut in their respective orientations to the crystal axis before being machined and completed to the required sizes.

Due to its acoustic sensing principle, which has been utilized to monitor cell apoptosis-induced viscoelastic properties, a QCM biosensor measurement satisfies the requirements of real-time and non-invasive analysis [14]. However, it is still challenging to distinguish different modalities of cell death based just on their viscoelastic properties. In this study, we presented the DRPC (Double resonator piezoelectric cytometry) technology, which is based on the piezoelectric principle of QCM but uses both AT and BT cut quartz crystals to quantify the cells-generated force. EerNisse pioneered this dual resonator technology [15]. As the silicon dioxide quartz crystals are anisotropic in that their physical properties are different along different molecular axes of the crystal, AT and BT cut quartz crystal oscillate in a shear mode with identical stress constants but opposing signs. Schematic illustrations of AT and BT cut crystals are shown in Scheme 1. A frequency shift with opposing sign but with the same magnitude is caused by the same lateral stress in AT and BT cut. This technology has been applied to measure the stress induced by hydrogen insertion in LaNi_5_ [16] and to measure the stress generated in situ during the electrodeposition of a Cu/Fe_20_Ni_80_ multilayer [17] and the progress of the lithium transportation [18]. However, it is not clear if it is also applicable to living cells of viscoelastic feature; in our previous work [19], we presented the theoretical background and equations to quantify cells’ generated forces and viscoelasticity using the DRPC technology. In this study, the DRPC technology was used for the first time to quantify cells-generated stress (Δ*S*) in identifying pyroptosis and apoptosis in real-time while simultaneously monitoring cells viscoelasticity as represented by the elastic/storage modulus (*G*′) and the viscous/loss modulus (*G*″). In addition, a specialized HeLa cell line deficient in GSDMD with an engineered -DEVD- sequence (GSDMD^−/−^-DEVD-) was utilized to testify pyroptosis and non-pyroptosis-induced mechanical response. 

## 2. Materials and Methods

### 2.1. Cell Culture and Reagents

HeLa were obtained from ATCC, GSDMD^−/−^-DEVD- mutant expressing HeLa cells were gifts from the laboratory of Professor Feng Shao (National Institute of Biological Sciences, Beijing, China), and both were grown at thermostatic 37 °C in DMEM (Gibco, Thermo Fisher Scientific, Waltham, MA, USA) supplemented with 10% fetal bovine serum (Gibco, Co., Waltham, MA, USA) in an atmosphere containing 5% (*v*/*v*) CO_2_ and 10% in humidified incubator. HeLa cells were transfected with constructs encoding green fluorescent protein to tag integrin and vinculin. Lipopolysaccharides (LPS), tumor necrosis factors α (TNF-α), and cycloheximide (CHX) were purchased from Sigma-Aldrich. Cholera toxin B subunit (CTB) was purchased from Absin, China.

### 2.2. Preparation and Modification of Electrode Surfaces of Quartz Crystals

The exposed gold electrode portion was placed with a drop of piranha solution (1:3 mixture of 30% H_2_O_2_ and 98% H_2_SO_4_) heated to 80 °C for 30 s, followed by rinsing with Milli-Q water and ethanol, then drying with ultra-high purity N_2_. This procedure was repeated three times. The quartz crystal was then sandwiched between two silicon O-rings in a vertical laminar hood using a Teflon well holder. Afterwards, the piranha-treated gold surface was exposed to absolute alcohol solution containing 20 mM 3-mercaptopropionic acid (MPA) and 1 mM triethylene glycol mono-11-mercaptoundeycl ether (TGME) for 16 h in the dark at room temperature to create a self-assembled monolayer (SAM). The gold surface coated with SAM was then immersed for 60 min in a phosphate buffer solution (PBS, pH = 5.5) containing 150 mM EDC and 30 mM N-hydroxysuccinimide (NHS) to attach the NHS group to the -COOH terminus of SAM. The substrates were then incubated for 6 hours at room temperature with fibronectin (20 µg/mL) in PBS (pH = 8.2). After each step of surface modification, the substrate was rinsed with its original solvent and Milli-Q water, respectively, to remove any loosely bound moieties.

### 2.3. Inductions of Pyroptosis and Apoptosis

Following the previously outlined protocol, 2000 HeLa cells in a volume of 400 μL were seeded into each well and cultured in a 5% CO_2_ incubator at 37 °C for the duration of the experiment. A lab pipettor was used to remove supernatant of media with the same volume of media containing the drug to be added, followed by the injection with drug. Lipopolysaccharide (LPS) and cholera toxin B (CTB) were used to induce pyroptosis; CTB worked as a deliverer, transporting LPS into the cell cytoplasm [20,21]. Specifically, 20 µL of DMEM media was removed from each well and replaced with 20 µL of LPS (1 μg/mL) for the first stimulation; six hours later, the same procedure was done but with 20 µL of LPS (100 ng/mL) and 20 µL of CTB (10 μg/mL) to enhance pyroptosis efficacy. To induce HeLa apoptosis, tumor necrosis factor family with a cognate membrane receptor (TNF-α) and cycloheximide (CHX) were used in combination. An amount of 40 µL of TNF-α (20 ng/mL) and 20 µL of CHX (10 μg/mL) were utilized to induce apoptosis. For GSDMD^−/−^-DEVD- mutant expressing HeLa pyroptosis and non-pyroptosis induction, 40 µL of TNF-α (20 ng/mL) and 20 µL of CHX (10 μg/mL) were utilized to induce GSDMD^−/−^-DEVD- HeLa pyroptosis. LPS and CTB were unable to induce pyroptosis in this case. 

### 2.4. Fabrication and Measurement of DRPC Based on QCM and 250B-Network Analyzer

**Scheme 2:**
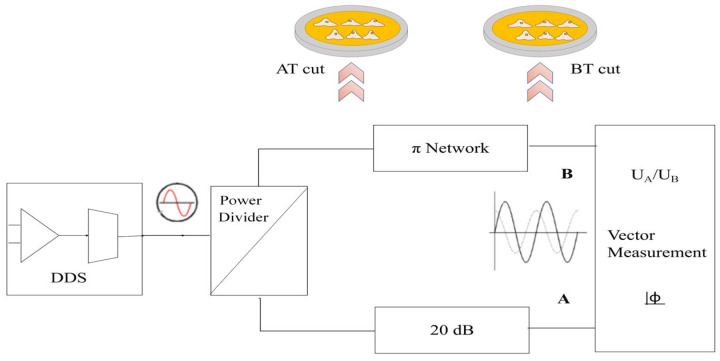
Illustration of the principle of measurement. DDS (Direct digital frequency synthesizer) generated the required scanning frequency, then the signal was split into two parts by the power divider. One was sent to channel A of the vector measurement unit with 20 dB attenuation as the reference signal, and the other was sent to channel B of the vector measurement unit through the π-Network. The quartz crystal resonators were connected into the πNetwork. The vector measure unit measures the voltage ratio and the phase difference between the two channels. Then, the frequency shift, motional resistance, motional inductance, and static capacitance were determined by the special algorithm of the software.

An amount of 9 MHz AT and BT cut quartz crystal resonators with a diameter of 12.5 mm and a thin layer of 100 nm gold electrode (5 mm diameter) were employed. The building material for the well was Teflon, a thermoplastic material with solvent inertia and hydrophobicity property with an additional feature that proteins and cells do not adhere to Teflon. Through thermo-molding process, duplicate Teflon wells can be produced. The matched Teflon well holders were used to support the quartz crystal resonators. The 250B Network Analyzer was purchased from Saunders & Associates (Phoenix, AZ, USA) with specially designed software for 4 channel quartz crystals tests. Detailed illustrations of the measure principle are given in Scheme 2.

**Scheme 3:**
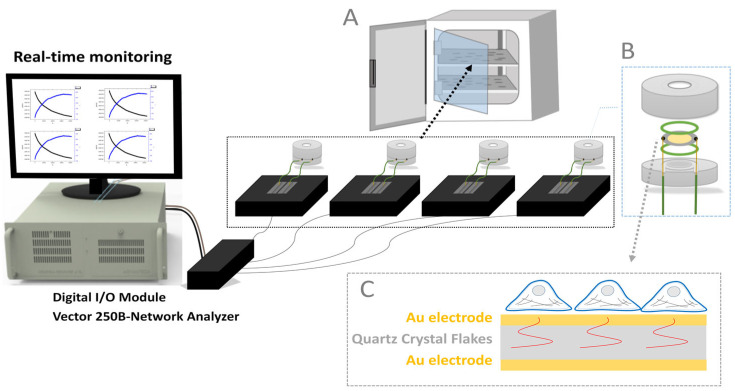
Schematic illustration of the fabrication of QCM-based DRPC and the measurement principle. (**A**) The measurement component and Teflon wells can be placed in an incubator set to 37 °C, 5% CO_2_, allowing four channels to operate concurrently. (**B**) The exploded-view of a Teflon well, AT and BT cut quartz crystals were held in a Teflon well with two tinned copper wires that were PVC-wrapped (polyvinyl chloride). The tinned copper was then inserted into an electrical conductor that was connected to the 250B network analyzer. (**C**) The side-view of cells adhesion on quartz crystal-Au electrode.

The fabrication of the QCM-based DRPC was schematically illustrated in Scheme 3, 400 μL of DMEM media containing 2000 HeLa cells and 10% FBS was added to each well equipped with a fibronectin-modified QCM gold electrode substrate. QCM frequency shift and motional resistance were collected continuously at a sampling rate up to one set per second. Throughout the experimental process, the entire module was kept at a 37 °C, 5% CO_2_ incubator and connected to the 250B-2 Vector Network Analyzer, allowing four channels to operate concurrently. Origin 8.0 software was used to process and plot the data. The fabrication of the QCM-based DRPC was schematically illustrated in Scheme 3.

## 3. Results

### 3.1. Theoretical Background and Equations for DRPC

The detailed theoretical background and equations for DRPC were presented in previous work [19]. Here, we introduced this technology briefly. Over the last two decades, QCM has garnered considerable attention in the biological field, particularly for monitoring the dynamic adhesion between cell and substrate due to its unique real-time, non-invasive, label-free acoustic sensing capability. The frequency shift (Δ*f*) and motional resistance (Δ*R*) or energy dissipation (Δ*D*) are key QCM parameters in monitoring dynamic adhesion process of cells. The frequency shift during the adhesion of a population of living cells to the quartz crystal without obvious cell–cell interactions is attributed to the following three variables of the cells: the mass, the stress exerted, and the viscoelasticity, which can be described as follows:

(1) $$\Delta f/f_{0}=\Delta f_{s}/f_{0}+\Delta f_{m}/f_{0}+\Delta f_{visco}/f_{0}$$

Therein, the frequency shift induced by cells’ exerted mechanical stress (Δ*f_s_*) is:

(2) $$\Delta f_{s}/f_{0}=K\Delta S/t_{q}$$

where Δ*S* is the lateral stress exerted by the cells, *K* is the cut-dependent lateral stress constant, and *t_q_* is the thickness of the crystal. 

To exclude the effects of living cells’ mass and viscoelasticity, the mass-induced frequency (Δ*f_m_*) has been subtracted, which is:

(3) $$\Delta f_{m}/f_{0}=-2f_{0}\Delta m\sqrt{\rho_{q}\mu_{q}}$$

where $\rho_{q}$ is the density of quartz crystal; *μ_q_* is the elastic modulus of quartz crystal, which is cut dependent.

The frequency shift induced by viscoelasticity of the cells (Δ*f_visco/cells_*) can be regarded as the viscoelasticity-induced frequency changes relative to the air (Δ*f_visco/air_*) minus the frequency induced by cell culture media (Δ*f_media_*), which is [19,22]:

(4) $$\Delta f_{visco/cells}/f_{0}=\Delta f_{visco/air}-\Delta f_{media}=-\frac{\sqrt{\frac{\rho_{c}}{2}\left(\left|G^{*}\right|-G^{\prime }\right)}}{\pi \sqrt{\rho_{q}\mu_{q}}}-\frac{\Delta f_{media}}{f_{0}}$$

where |G*| is the absolute value of the complex modulus, *G*′ is the storage modulus of the cells, and *ρ_c_* is the cells’ density.

Together, the total frequency shifts induced by the living cells is:

(5) $$\;\;\Delta f/f_{0}=K\Delta S/t_{q}-{\left(\sqrt{\rho_{q}\mu_{q}}\right)}^{-1}\left(2f_{0}\Delta m+\pi^{-1}\sqrt{\rho_{c}\left(\left|G^{*}\right|-G^{\prime }\right)/2}\right)$$

When subjected same stress to *AT* and *BT* cut quartz crystals, it would exhibit same magnitude of frequency shift but with the opposite sign. For *AT* and *BT* cut crystals of the same frequency and surface conditions, it can be assumed that both crystals would adhere the same amount and quality of cells with identical mass, viscoelasticity, and exerted stress, and Equation (5) can then be applicable to both *AT* and *BT* cut crystals. Then, the lateral stress (Δ*S*) generated by cells at living cells/quartz crystal interface can be determined with the following equation:

(6) $$\;\Delta S_{t}=f_{0}^{-1}{\left(K^{AT}-K^{BT}\right)}^{-1}\left(\Delta f_{t}^{AT}t_{q}^{AT}-\Delta f_{t}^{BT}t_{q}^{BT}\right)$$

where *K^AT^* = 2.75 × 10^−12^ cm^2^ dyn^−1^, *K^BT^* = −2.65 × 10^−12^ cm^2^ dyn^−1^ are quartz crystal stress constants; *t_q_^AT^* and *t_q_^BT^* are quartz crystal thicknesses in cm, *f*_0_*^AT^* and *f*_0_*^BT^* are the original resonant frequency in Hz, and *f_t_^AT^* and *f_t_^BT^* are the frequency shifts generated when resonators experience the surface stress effects minus the respective *f*_0_ at time *t*. 

When *f*_0_*^AT^* = *f*_0_*^BT^* = 9 MHz, the above Equation (6) can be further simplified to:

(7) $$\Delta S=380.8\Delta f^{AT}-582.2\Delta f^{BT}$$

Positive value (Δ*S* > 0) indicates the tension in thin film on interface [18]. In our work, tension is generated by cell contractile force/traction force in maintaining its adhesion on the substrate while the quartz crystal surface is under compressive stress. In contrast, negative value (Δ*S* < 0) indicates the protrusive force dominating on the substrate that leads to tensile stress in the quartz crystal.

In addition, the DRPC technology permits simultaneous monitoring of cell viscoelasticity in a real-time manner. The elastic modulus (storage modulus) *G*′ and the viscous modulus (loss modulus) *G*″ are determined through the measured frequency shifts Δ*f* (subtracted from the stress-induced frequency shift) and the half bandwidth or motional resistance change *R* relative to their values in air, which are shown as:

(8) $$G^{\prime }=\pi^{2}Z_{q}^{2}{\left(\rho_{c}f_{0}^{2}\right)}^{-1}\left(\Delta R^{2}∕16\pi^{2}L_{q}^{2}-\Delta f^{2}\right)$$

(9) $$G^{\prime \prime }=-\pi Z_{q}^{2}\Delta f\Delta R{\left(2\rho_{c}L_{q}f_{0}^{2}\right)}^{-1}$$

where the *Z_q_* is the impedance of quartz crystal, *Z_q_^AT^* = 8.84 × 10^5^ g/cm•s, *Z_q_^BT^* = 1.35 × 10^6^ g/cm•s, *L_q_* is the measurable motional inductance of quartz crystal immersed in media, which can be regarded as constant.

### 3.2. Cytomechanical Dynamics during HeLa Pyroptosis and Apoptosis

**Figure 1:**
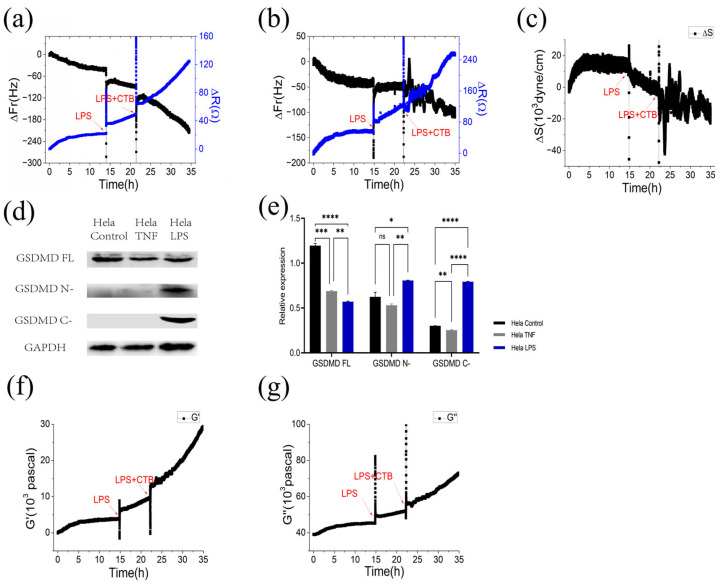
Dynamic QCM responses and cytomechanical parameters recorded during LPS and CTB induced pyroptosis in 2000 HeLa cells. (**a**,**b**) Frequency shifts and motional resistance changes on AT (**a**) and BT (**b**) cut quartz crystal; (**c**) Cells generated force on quartz crystal; (**d**) Effects of pyroptosis induced by LPS and CTB on GSDMD cleavage through Western blot assay. The loading control is GAPDH expression; (**e**) The relative expression of target protein/GAPDH was analysed by Image-Pro Plus 6.0 Software (* *p* < 0.05, ** *p* < 0.01, *** *p* < 0.001, **** *p* < 0.0001); (**f**) Variations of the elastic modulus (storage modulus) *G*′; (**g**) Variations of the viscous modulus (loss modulus) G″.

The induction of pyroptosis by LPS and CTB was confirmed by the Western blot assay through detecting the cleaved GSDMD protein product N-terminal (pore-forming protein) and C-terminal (Figure 1), which was considered the pyroptotic marker [23]. Figure 1 showed the time course of QCM responses (frequency shift Δ*f* and motional resistance Δ*R*) on *AT* and *BT* cut quartz crystals of 2000 HeLa cells, as well as the changes in cells-exerted stress (Δ*S*), storage modulus (*G*′), and loss modulus (*G*″) upon LPS and CTB stimulation. During the adhesion process, the cells generated nearly 20 × 10^3^ dyne/cm stress on quartz crystal, which gradually became stable. After LPS treatment, the cells-induced QCM response exhibited a decrease in Δ*f* and an increase in Δ*R* on both AT and BT cut, as well as a change in the value of Δ*S* from positive to negative. Six hours later, CTB was combined with LPS to enhance the efficacy of pyroptosis induction, the co-stimulus induced a more significant decrease in Δ*f* and an increase in Δ*R*, as well as a further decrease in Δ*S*. Notably, pyroptosis has changed the sign of Δ*S* value (from positive to negative), suggesting that pyroptosis altered the cells-exerted stress on quartz crystal. At initial stage of cell spreading and adhesion, cell traction force is dominant, cells exert compressive stress on substrate to mediate its adhesion [24]. In this stage, Δ*S* maintained at a positive value and gradually rose with the mature of FA, when cell spreading area and cell traction force eventually become constant, Δ*S* turned to be stable. However, we discovered that during pyroptosis, the compressive stress exerted by cells on quartz crystal transforms into a tensile stress, and Δ*S* changes sign from positive to negative. According to the cell tensegrity model, the disruption of cytoskeletal structure during pyroptosis is responsible for the transition in cells-exerted stress, which is primarily attributed to the disruption of intermediate filaments that affects the generation of cell traction force, thereby resulting in the predominance of protrusive force [25]. In addition, microtubule forces involved in the development of membrane blebs during pyroptosis are also linked to the change of the dominant force [26]. By combining with the viscoelastic responses, a far more detailed picture of the pyroptosis mechanical properties has been presented. The storage modulus (*G*′) and the loss modulus (*G*″) increased in response to the first LPS treatment and the second co-treatment with LPS and CTB. As the elastic modulus is correlated with cytoskeletal changes [27], it is likely that the F-actin polymerization or retrograde actin flow that drives the plasma membrane outward during pyroptosis leads to an increase in the elastic modulus.

**Figure 2:**
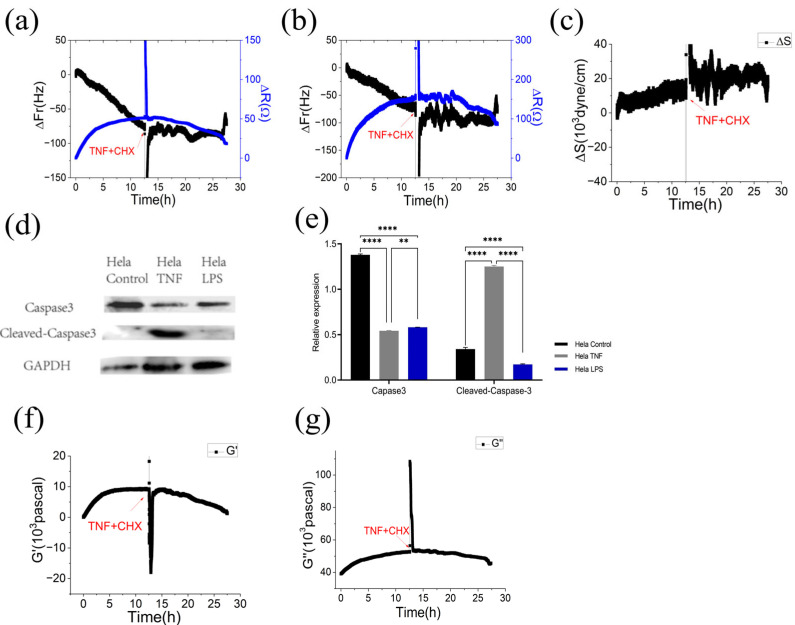
Dynamic QCM responses and cytomechanical parameters recorded during TNF and CHX induced apoptosis in 2000 HeLa cells. (**a**,**b**) Frequency shifts and motional resistance changes on AT (**a**) and BT (**b**) cut quartz crystals; (**c**) Cells generated force on quartz crystal; (**d**) Effects of apoptosis induced by TNF and CHX on Caspase3 cleavage through Western blot assay. The loading control is GAPDH expression; (**e**) The relative expression of target protein/GAPDH was analysed by Image-Pro Plus 6.0 Software (** *p* < 0.01, **** *p* < 0.0001); (**f**) Variations of the elastic modulus (storage modulus) *G*′; (**g**) Variations of the viscous modulus (loss modulus) *G*′.

In contrast, distinct QCM responses were observed in apoptosis (Figure 2). The Δ*f* value slightly increased as ΔR decreased, which is opposite with pyroptosis and consistent with prior investigations [28]. In addition, the Δ*S* value increased and peaked at around 40 × 10^3^ dyne/cm after TNF and CHX treatment, demonstrating that apoptotic cells exert higher compressive stress on quartz crystal. According to the cell tensegrity model [25], cell prestress is balanced by inter-connected structural features that resist compression, such as internal microtubules, and external traction on the ECM. In apoptosis, the inner compression element microtubules that maintain the cell’s shape are disrupted, resulting in the loss of prestress function [29]. With the depolymerization of microtubules, the stored stress and energy are transferred to another balance structure, the external extracellular matrix (ECM), resulting in an increase in traction force [30,31,32]. Similarly, declining *G*′ and *G*″ were observed in apoptosis, consistent with prior studies [12,33]. It is believed that the depolymerization of F-actin, the disruption of actin fibers in the cytoskeleton, and the absence of F-actin filaments are responsible for the decreasing elastic modulus [34,35], demonstrating that traction force enhancement is accompanied by the intercellular structure reorganization in apoptosis.

**Figure 3:**
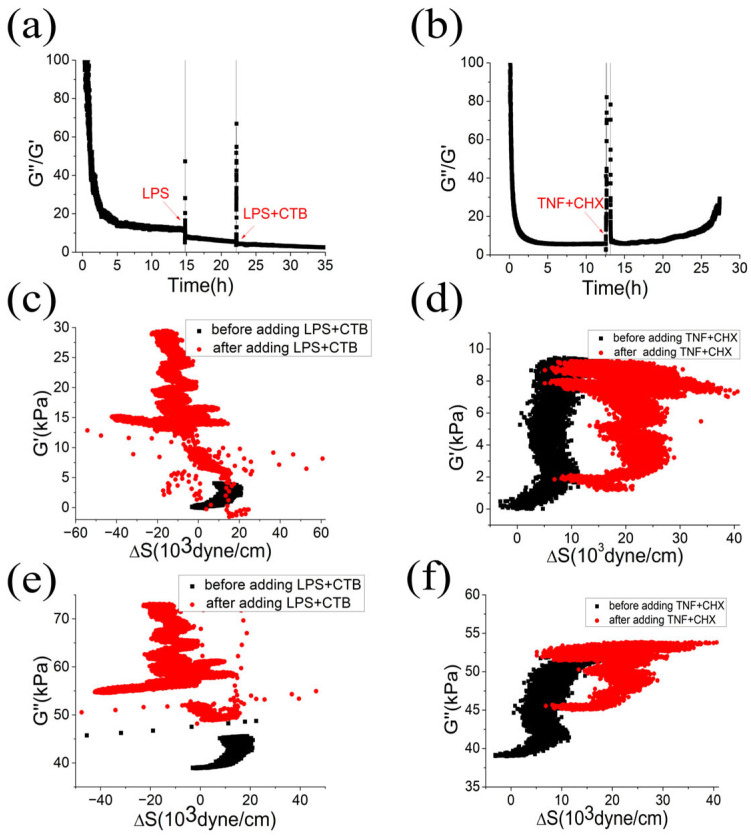
Changes in loss tangent and correlations between cytomechanical parameters during the LPS and CTB induced pyroptosis and TNF and CHX induced apoptosis in 2000 HeLa cells on FN modified quartz crystal-Au surfaces. (**a**,**b**) changes in loss tangent η = *G*″/*G*′; (**c**) *G*′ versus Δ*S* correlation during LPS+CTB stimulation; (**d**) *G*′ versus Δ*S* correlation during TNF+CHX stimulation; (**e**) *G*″ versus Δ*S* correlation during LPS+CTB stimulation; (**f**) *G*″ versus Δ*S* correlation during TNF+CHX stimulation.

Comparing *G*′ and *G*″ during cell adhesion stage (10–14.5 h) to pyroptosis/apoptosis stage (24–27 h), it can be observed that *G*′ has increased nearly five-fold during pyroptosis and decreased almost 5 kPa during apoptosis while *G*″ has increased nearly 15 kPa during pyroptosis and decreased 5 kPa during apoptosis (Supplementary Table S2). To dig out more information about cell viscoelasticity in pyroptosis and apoptosis, the loss tangent *G*″/*G*′ (the ratio of viscous modulus over elastic modulus) is plotted against time in Figure 3. It presented the relative contributions of *G*″ and *G*′ and is interpreted as a tendency to solid-like (loss tangent decreased) or fluid-like (loss tangent increased) cell behavior. During the adhesion process, cells exhibited a rapid change from a fluid-like state (loss tangent >> 100) to a viscoelastic gel state (loss tangent < 10) and remained stable. In pyroptosis, the loss tangent decreases, whereas in apoptosis, the loss tangent rises. As previously reported [12], the active reorganization and degradation of the cytoskeleton during apoptosis is attributed to the rising loss tangent. In contrast, the decreasing loss tangent observed in pyroptotic cells suggests a change from fluid-like (solution) to solid-like (gel). 

To summarize, the mechanical responses during pyroptosis and apoptosis exhibited substantial distinctions. Specifically, Δ*f* reduced in pyroptosis but elevated in apoptosis, whereas Δ*R* elevated in pyroptosis but reduced in apoptosis. In addition, the Δ*S* value exhibited a “positive-negative” transition in pyroptosis, indicating that the protrusive force that stresses the quartz crystal became the dominant force during pyroptosis process, whereas the traction force continues to dominate in apoptotic cells, resulting in an increase in Δ*S*. In addition, the elastic and viscous moduli exhibited an upward trend in pyroptosis and a downward trend in apoptosis, whereas the loss tangent reduced in pyroptosis and increased in apoptosis. Notably, this is the first time that a “sol-gel” transition has been discovered during pyroptosis. Then, we compared the key components of cell adhesion complexes in pyroptosis and apoptosis in order to figure out the relationship between mechanical transition and cell adhesion state in these two forms of cell death.

### 3.3. Cytoskeletal Changes during HeLa Pyroptosis and Apoptosis

**Figure 4:**
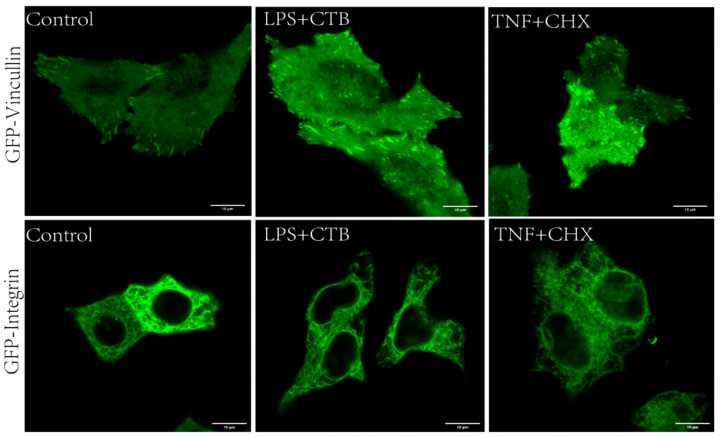
The expressions of integrin and vinculin in pyroptosis and apoptosis. HeLa cells were transfected with GFP-vinculin and GFP-integrin constructs. The upper row depicted the vinculin expressions in control group in response to LPS and CTB, TNF, and CHX stimulations. The lower row depicted the integrin expressions in control group in response to LPS and CTB, TNF, and CHX stimulations.

Cell morphology and cell adhesion status altered differentially in pyroptosis and apoptosis, with pyroptosis characterized primarily by cell swelling and volume enlargement and apoptosis by cell shrinkage and blebbing. Alterations in morphology and cell adhesion are the result of alterations in the cytoskeleton’s structure. However, little is known regarding the relationship between pyroptosis and apoptosis’ internal cytoskeletal alterations, external cell adhesion status, and cell mechanical changes. Thus, we investigated the essential internal and external components of cells during pyroptosis and apoptosis. Integrin served as the key transmembrane protein in mediating the cell-extracellular matrix (ECM) adhesion and transmitting intrinsically-generated and extrinsically-sensed mechanical or chemical signals inside-out and outside-in. Force transmission is accomplished through the physical connection of the ECM and intracellular actin cytoskeleton via vinculin, talin, and other related binding proteins [36,37]. Vinculin is recognized as the primary link between integrins and actin among these proteins. Therefore, we compared the expression changes of internal vinculin and external transmembrane protein integrin in pyroptosis and apoptosis. The observed of GFP fluorescence with laser scanning confocal microscopy (LSCM) was presented in Figure 4. 

In the control group, vinculin was predominantly located at the cell periphery and displayed in a streak-like pattern, as depicted in Figure 4. Upon stimulation with LPS and CTB, the streak-like vinculin at the cell periphery and cell–cell contact edges remained distinct, and small dot-like vinculin formed at cell periphery. In contrast, streak-like vinculin nearly disappeared after TNF and CHX stimulation. Previous research has shown that the absence of vinculin during apoptosis is due to actin stress fiber disorganization, which affects the organization of the cytoskeleton and decreases the elastic modulus [38]. According to our findings, the absence of vinculin in HeLa apoptosis decreases both the elastic and viscous moduli. Specifically, the decreasing *G*″/*G*′ indicates the elastic modulus dominating state in pyroptosis, whereas the disappearance of vinculin results in the increasing *G*″/*G*′, a viscous modulus dominating state in apoptosis, indicating that the cell viscoelastic state is partially related to the vinculin expression level. 

Integrins form a compacted pattern that encircles the cell body in the control, then a wide spreading, anisotropic elongated pattern following stimulation with LPS and CTB, but a dissipated, dispersed pattern with decreased expression after stimulation with TNF and CHX. Combining with viscoelastic responses, the increased elastic and viscous modulus during pyroptosis corresponds to the maintaining of vinculin expression and newly-formed vinculin. As the expression of vinculin is tightly related to the quantities and spreading degree of FAs [39], the decrease in the expression of integrins is the chain reaction of vinculin loss. The loss of integrins causes the loss of ECM-integrins linking, which decreases *G*″ in apoptosis, but newly-formed integrins increase the ECM-integrins linking, resulting in an increase in *G*″ in pyroptosis. As the spreading of integrin in pyroptosis corresponds to its firm attachment on a substrate during the entire pyroptosis process until ultimate death [40], the increasing modulus in pyroptosis may be attributed to ECM-integrins linkage maintenance.

### 3.4. Cytomechanical Dynamics during HeLa GSDMD^−/−^-DEVD- Pyroptosis and Non-Pyroptosis

In order to rule out the influence of stimulation drugs and confirm the mechanics model for identifying HeLa pyroptosis and apoptosis, we investigated the mechanical response of GSDMD^−/−^-DEVD- mutant expressing HeLa cells. The mutant expressing HeLa cells combined the GSDMD-knockout with an engineered -DEVD- insertion sequence generated by CRISPR-Cas9 targeting. As the pyroptosis executive protein, GSDMD acted as the key in pyroptosis execution. In the absence of GSDMD, LPS and CTB stimulation fails to induce pyroptosis [23]. Additionally, the study discovered that inflammatory caspases cleave the GSDMD specifically at the _272_FLTD_275_ site, which is the only possible inflammatory caspase cleavage site that is conserved in both human and mouse GSDMD [41]. The -FLTD- site was engineered into the -DEVD- site (the caspase-3/7 cleavage site during apoptosis) and expressed in GSDMD^−/−^ HeLa cells, resulting in the switch from apoptosis to pyroptosis in response to TNF and CHX stimulation [23]. In combination, we have GSDMD^−/−^-DEVD- mutant expressing HeLa cells that are resistance to pyroptosis in response to LPS and CTB stimulation but execute pyroptosis in response to TNF and CHX stimulation.

**Figure 5:**
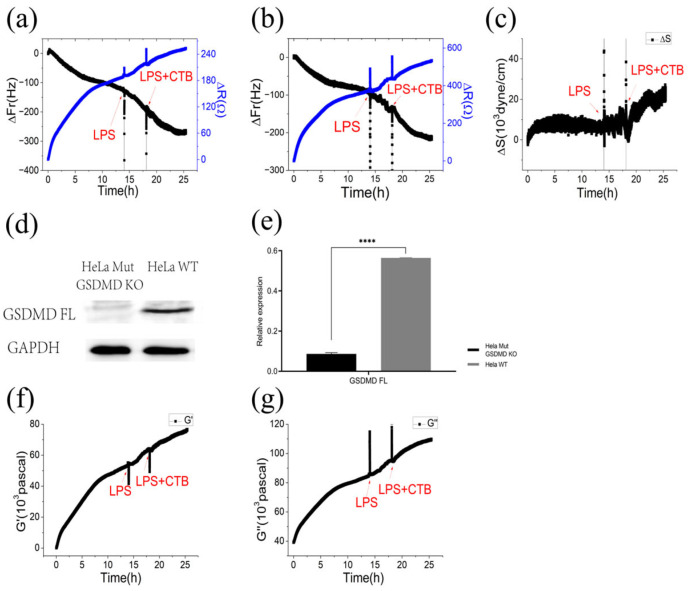
Dynamic QCM responses and cytomechanical parameters recorded during LPS and CTB induced pyroptosis-resistance in 2000 GSDMD^−/−^-DEVD- mutant expressing HeLa cells. (**a**,**b**) Frequency shifts and motional resistance changes on AT (**a**) and BT (**b**) cut quartz crystals; (**c**) Cells generated force on quartz crystal; (**d**) GSDMD knockout cells generated by CRISPR-CAS9-mediated targeting, and its expression is verified through Western blot assay. The loading control is GAPDH expression; (**e**) The relative expression of target protein/GAPDH was analysed by Image-Pro Plus 6.0 Software (**** *p* < 0.0001); (**f**) Variations in the elastic modulus (storage modulus) *G*′; (**g**) Variations in the viscous modulus (loss modulus) G″.

The non-pyroptosis QCM responses (stimulated with LPS and CTB in GSDMD^−/−^-DEVD-) are identical to HeLa pyroptosis responses (stimulated with LPS and CTB in HeLa), with frequency decreasing and resistance increasing (Figure 5). Nevertheless, the Δ*S* response to LPS and CTB treatment in GSDMD^−/−^ -DEVD- cells was unable to cause force direction change; the Δ*S* fluctuated erratically after the initial LPS stimulation and increased considerably after LPS and CTB co-stimulation, indicating that protrusive force is predominant only in pyroptotic cells. Additionally, the elastic modulus (*G*′) and loss modulus (*G*″) increased concurrently in the non-pyroptosis case.

**Figure 6:**
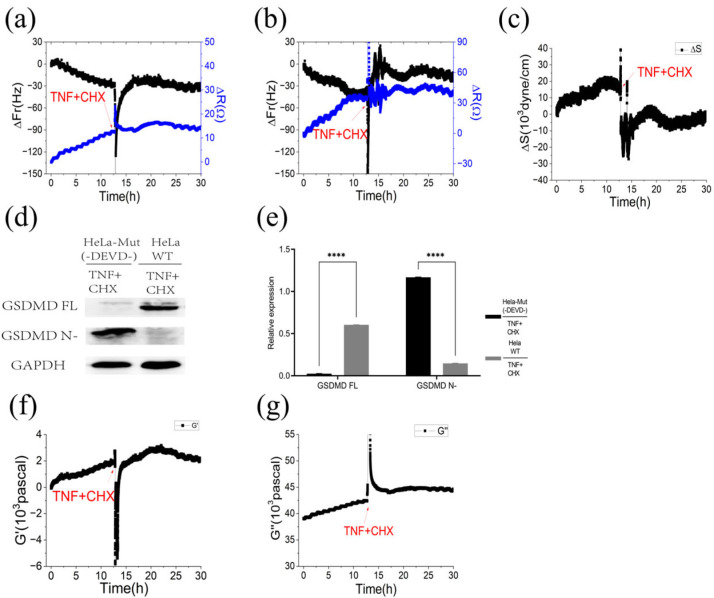
Dynamic QCM responses and cytomechanical parameters recorded during TNF and CHX induced pyroptosis in 2000 GSDMD^−/−^-DEVD- mutant expressing HeLa cells. (**a**,**b**) Frequency shifts and motional resistance changes on AT (**a**) and BT (**b**) cut quartz crystals; (**c**) Cells generated force on quartz crystal; (**d**) Switch of TNF induced apoptosis to pyroptosis by caspase-3 sensitive GSDMD mutant. GSDMD-DEVD- (replacement of the FLTD motif with the caspase-3-cleavage site DEVD) was stably expressed in HeLa GSDMD^−/−^cells. Cells were treated with TNF and CHX for 8 h, GSDMD was proteolytically cleaved into specific size, GSDMD N- can then be detected upon TNF and CHX stimulation through Western Blot assay. GAPDH expression is the loading control; (**e**): The relative expression of target protein/GAPDH was analysed by Image-Pro Plus 6.0 Software (**** *p* < 0.0001); (**f**) Variations in the elastic modulus (storage modulus) *G*′; (**g**) Variations in the viscous modulus (loss modulus) G″.

In another group, TNF and CHX were used to induce pyroptosis in mutant expressing HeLa cells. In this case of pyroptosis (Figure 6), the QCM responses were noticeably distinct from those of the HeLa pyroptosis, with both Δ*f* and ΔR increasing despite the fact that the Δ*f* increased significantly higher in the BT cut than in the AT cut. Therefore, Δ*S* response was observed to have dropped from a positive value to a negative value. Additionally, the elastic modulus (*G*′) and viscous modulus (*G*″) both increased in this case of pyroptosis, which is consistent with HeLa pyroptosis, although in this case, the growth rate of pyroptosis is significantly lower than HeLa pyroptosis. 

**Figure 7:**
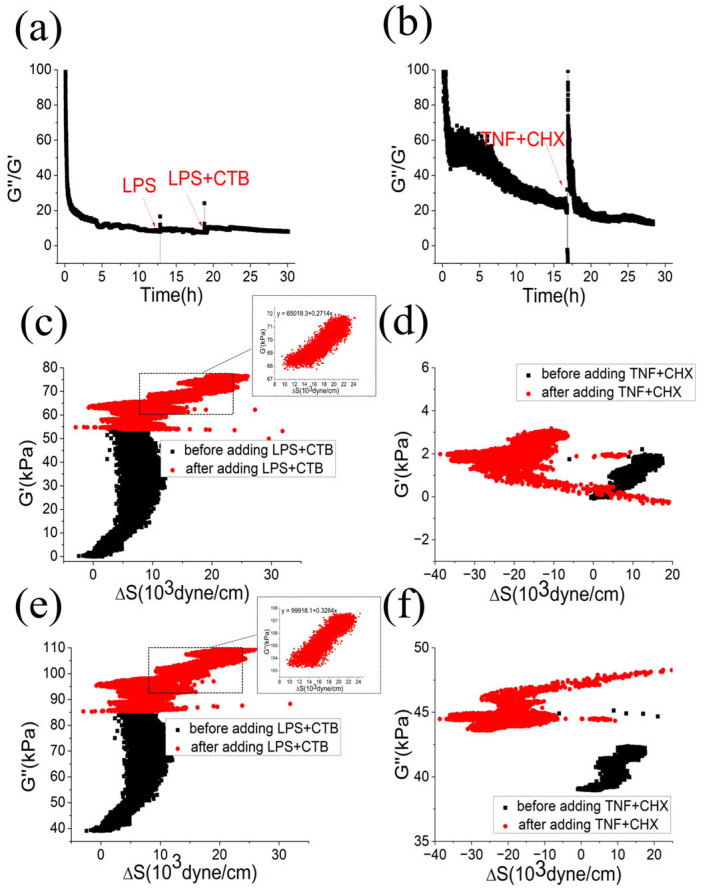
Changes in loss tangent and correlations between cytomechanical parameters during the LPS and CTB induced pyroptosis-resistance and TNF and CHX induced apoptosis in 2000 GSDMD^−/−^(-DEVD-) mutant expressing HeLa cells on FN modified quartz crystal-Au surfaces. (**a**,**b**) changes in loss tangent η = *G*″/*G*′; (**c**) *G*′ versus Δ*S* correlation during LPS+CTB stimulation; (**d**) *G*′ versus Δ*S* correlation during TNF+CHX stimulation; (**e**) *G*″ versus Δ*S* correlation during LPS+CTB stimulation; (**f**) *G*″ versus Δ*S* correlation during TNF+CHX stimulation.

Although the elastic modulus and viscous modulus increased in both LPS+CTB induced non-pyroptosis and TNF+CHX-induced pyroptosis, distinct differences were observed when comparing the loss tangent (*G*″/*G*′) in these two cases (Figure 7), the loss tangent remained unchanged in non-pyroptosis case, but decreased slightly in GSDMD^−/−^-DEVD- HeLa pyroptosis case. This result indicates that the “sol-gel” transition also occured in this pyroptosis case. To summarize, the mechanical responses in both HeLa and GSDMD^−/−^-DEVD- HeLa pyroptosis are highly consistent with a sign transition inΔ*S*, an increase in *G*′, G″, and a decrease in *G*″/*G*′. Moreover, Δ*S* value tripled in GSDMD^−/−^-DEVD- HeLa non-pyroptosis (Supplementary Table S3), which was similar with the HeLa apoptosis response. The distinction lies in their viscoelastic responses, with *G*′ and *G*″ increasing and *G*″/*G*′ remaining unchanged in the GSDMD^−/−^-DEVD- HeLa non-pyroptosis case. Through AFM technology, these viscoelastic features have been observed in extrinsic apoptosis and were ascribed to the reorganization of cytoskeletal components without destroying the cytoskeletal polymer [12]. It is likely that LPS and CTB possibly induced an extrinsic apoptosis in GSDMD^−/−^-DEVD- HeLa cells. In addition, we identified the approximately linear correlations between Δ*S*-*G*′ and Δ*S*-*G*″ in GSDMD^−/−^-DEVD- non-pyroptosis case (Figure 7c,e). However, no linear correlation was observed in any of the other three cases of cell death. The approximately linear correlations between cells’ traction force and cells’ viscoelastic modulus occur in situations where cytoskeleton is comparatively intact, according to cell tensegrity model [25]. Thus, the linear correlation in the non-pyroptosis case of GSDMD^−/−^-DEVD- may be attributable to the ineffective cell death induction, the cytoskeleton of GSDMD^−/−^-DEVD- HeLa was not evidently disrupted in comparison to the other three cases of cell death inductions.

## 4. Discussion

In the field of cell death detection, it still takes days for protein expressions or hours for biochemical signaling transmissions to identify, but mechanical transmissions only take minutes. However, most mechanical measurement techniques have limitations in terms of end-point assays and single-cell analysis, limiting their widespread in identifying cell death. In this study, we introduced a novel method—the DRPC technology—that enables the identification of cell pyroptosis and apoptosis in a real-time and non-invasive manner. Through measuring the cells’ generated force, clearly distinction can be observed in pyroptosis and apoptosis. The different Δ*S* responses (dominant force) of cells is the major mechanical distinction. In pyroptosis, the protrusive force that drives cell swelling predominates, resulting in a transition from positive to negative Δ*S* value, whereas in apoptosis, the traction force predominates, resulting in an increase in Δ*S* value without a change in sign. In other words, the underlying quartz crystal is subjected to tensile stress during pyroptosis but compressive stress during apoptosis. On the other hand, the viscoelastic properties in pyroptosis and apoptosis also differ substantially. We found that pyroptotic cells have increased elastic and viscous modulus, but reduced loss tangent (*G*″/*G*′), indicating that pyroptotic cells exhibited a “fluid-solid” transition. In contrast, apoptosis resulted in a decrease in both elastic and viscous moduli, which is consistent with prior studies and is believed to be the outcome of cytoskeleton degradation/depolymerization [12,42]. Moreover, the rising loss tangent (*G*″/*G*′) in apoptosis, suggesting that apoptotic cells exhibited a more fluid-like tendency. Afterwards, we performed the engineered GSDMD^−/−^-DEVD- expressing HeLa cells in order to rule out drug interference. LPS and CTB were incapable of inducing pyroptosis in this case, whereas TNF and CHX were effective inducers of pyroptosis. The results showed that the stress (Δ*S*), elastic modulus (*G*′), and viscous modulus (*G*″) responses in TNF+CHX induced pyroptosis are consistent with LPS+CTB induced HeLa pyroptosis, however the former growing rate is considerably lower than latter. The following variables may account for the difference between two cases of pyroptosis: (1) GSDMD are shown to serve a dual role in pyroptosis, not only executing pyroptosis but also promoting its amplification, cells without GSDMD may have poor pyroptosis induction efficiency [43]. (2) The GSDMD^−/−^-DEVD- mutant HeLa may gradually lose its expression during the process of cell passage, leading in the deviation of the results. While, in LPS+CTB induced non-pyroptosis case, the stress (Δ*S*) increased, which avoid the drug interference in mechanical measurement and proved the viability of the DRPC method.

In conclusion, we presented dynamic mechanical parameters during both pyroptosis and apoptosis that might be regarded as a mechanical model for identifying pyroptosis and apoptosis. We proved the DRPC feasibility in identifying pyroptosis and apoptosis by using both HeLa and engineered mutant expressing HeLa cells, as well as its potential for the development of predictive mechanical model under different biological conditions. Due to its real-time, non-invasive measurement, multiple mechanical parameters capturing, and compatibility with conventional cell culture configurations, the DRPC technology is comparable in importance and significance to conventional biochemical detection. Its advantages provide insight in regulating pyroptosis and apoptosis through early intervention, as well as a novel target for pyroptosis- and apoptosis-associated diseases used for drug screening. In the near future, high-throughput channels of DRPC technology that provide parallel analysis of larger quantities of cells will be introduced to meet the needs of quantitative cell mechanics. 

## Data Availability

The authors confirm that the data supporting the findings of this study are available within the articles.

## Supplementary Materials

The following supporting information can be downloaded at: https://www.mdpi.com/article/10.3390/bios13030356/s1, Table S1: Methods for detecting both cell apoptosis and pyroptosis. Table S2: Mean values of mechanical parameters during HeLa pyroptosis and apoptosis; Table S3: Mean values of mechanical parameters during GSDMD^−/−^ -DEVD- HeLa non-pyroptosis and pyroptosis.

## References

[1] Chiong M., Wang Z.V., Pedrozo Z., Cao D.J., Troncoso R., Ibacache M., Criollo A., Nemchenko A., Hill J.A., Lavandero S. (2011). Cardiomyocyte death: Mechanisms and translational implications. Cell Death Dis..

[2] Jurasz P., Courtman D., Babaie S., Stewart D.J. (2010). Role of apoptosis in pulmonary hypertension: From experimental models to clinical trials. Pharmacol. Ther..

[3] Hanahan D., Weinberg R.A. (2000). The hallmarks of cancer. Cell.

[4] Hanahan D., Weinberg R.A. (2011). Hallmarks of cancer: The next generation. Cell.

[5] Wu D., Wang S., Yu G., Chen X. (2021). Cell Death Mediated by the Pyroptosis Pathway with the Aid of Nanotechnology: Prospects for Cancer Therapy. Angew. Chem..

[6] Wang Q., Wang Y., Ding J., Wang C., Zhou X., Gao W., Huang H., Shao F., Liu Z. (2020). A bioorthogonal system reveals antitumour immune function of pyroptosis. Nature.

[7] Ji N., Qi Z., Wang Y., Yang X., Yan Z., Li M., Ge Q., Zhang J. (2021). Pyroptosis: A New Regulating Mechanism in Cardiovascular Disease. J. Inflamm. Res..

[8] Ferreira A.C., Soares V.C., de Azevedo-Quintanilha I.G., Dias S.D.S.G., Fintelman-Rodrigues N., Sacramento C.Q., Mattos M., de Freitas C.S., Temerozo J.R., Teixeira L. (2021). SARS-CoV-2 engages inflammasome and pyroptosis in human primary monocytes. Cell Death Discov..

[9] Doitsh G., Galloway N.L., Geng X., Yang Z., Monroe K.M., Zepeda O., Hunt P.W., Hatano H., Sowinski S., Muñoz-Arias I. (2014). Cell death by pyroptosis drives CD4 T-cell depletion in HIV-1 infection. Nature.

[10] Wang M., Jiang S., Zhang Y., Li P., Wang K. (2019). The Multifaceted Roles of Pyroptotic Cell Death Pathways in Cancer. Cancers.

[11] Kupcho K., Shultz J., Hurst R., Hartnett J., Zhou W., Machleidt T., Grailer J., Worzella T., Riss T., Lazar D. (2019). A real-time, bioluminescent annexin V assay for the assessment of apoptosis. Apoptosis Int. J. Program. Cell Death.

[12] Van der Meeren L., Verduijn J., Krysko D.V., Skirtach A.G. (2020). AFM Analysis Enables Differentiation between Apoptosis, Necroptosis, and Ferroptosis in Murine Cancer Cells. iScience.

[13] Schierbaum N., Rheinlaender J., Schäffer T.E. (2019). Combined atomic force microscopy (AFM) and traction force microscopy (TFM) reveals a correlation between viscoelastic material properties and contractile prestress of living cells. Soft Matter.

[14] Zhou B., Hao Y., Wang Z., Wei P., Du L., Xia Q. (2022). Dynamical and noninvasive monitoring of curcumin effect on the changes in the viscoelasticity of human breast cancer cells: A novel model to assess cell apoptosis. Talanta.

[15] EerNisse E.P. (1972). Simultaneous thin-film stress and mass-change measurements using quartz resonators. J. Appl. Phys..

[16] Li Y., Cheng Y.T. (1996). Studies of metal hydride electrodes using an electrochemical quartz crystal microbalance. J. Electrochem. Soc..

[17] Chassaing E. (1997). In situ mass changes and stress measurements in Cu/Fe_20_Ni_80_ electrodeposited multilayers. J. Electrochem. Soc..

[18] Pyun S.-I., Go J.-Y., Jang T.-S. (2004). An investigation of intercalation-induced stresses generated during lithium transport through Li1−δCoO_2_ film electrode using a laser beam deflection method. Electrochim. Acta.

[19] Zhou T., Huang J., Xiong L., Shen H., Huang F., Li W., Peng H., Su Z., Pan W., Zhao J. (2023). Real-Time Quantification of Cell Mechanics and Functions by Double Resonator Piezoelectric Cytometry—Theory and Study of Cellular Adhesion of HUVECs. bioRxiv.

[20] Yang J., Zhao Y., Shao F. (2015). Non-canonical activation of inflammatory caspases by cytosolic LPS in innate immunity. Curr. Opin. Immunol..

[21] Kayagaki N., Wong M.T., Stowe I.B., Ramani S.R., Gonzalez L.C., Akashi-Takamura S., Miyake K., Zhang J., Lee W.P., Muszyński A. (2013). Noncanonical inflammasome activation by intracellular LPS independent of TLR4. Science.

[22] Marx K.A., Zhou T., Montrone A., McIntosh D., Braunhut S.J. (2005). Quartz crystal microbalance biosensor study of endothelial cells and their extracellular matrix following cell removal: Evidence for transient cellular stress and viscoelastic changes during detachment and the elastic behavior of the pure matrix. Anal. Biochem..

[23] Shi J., Zhao Y., Wang K., Shi X., Wang Y., Huang H., Zhuang Y., Cai T., Wang F., Shao F. (2015). Cleavage of GSDMD by inflammatory caspases determines pyroptotic cell death. Nature.

[24] Henry S.J., Chen C.S., Crocker J.C., Hammer D.A. (2015). Protrusive and Contractile Forces of Spreading Human Neutrophils. Biophys. J..

[25] Wang N., Naruse K., Stamenović D., Fredberg J.J., Mijailovich S.M., Tolić-Nørrelykke I.M., Polte T., Mannix R., Ingber D.E. (2001). Mechanical behavior in living cells consistent with the tensegrity model. Proc. Natl. Acad. Sci. USA.

[26] Chen T., Guo Y., Shan J., Zhang J., Shen X., Guo J., Liu X.M. (2019). Vector Analysis of Cytoskeletal Structural Tension and the Mechanisms that Underpin Spectrin-Related Forces in Pyroptosis. Antioxid. Redox Signal..

[27] Kwon S., Yang W., Moon D., Kim K.S. (2020). Comparison of Cancer Cell Elasticity by Cell Type. J. Cancer.

[28] Braunhut S.J., McIntosh D., Vorotnikova E., Zhou T., Marx K.A. (2005). Detection of apoptosis and drug resistance of human breast cancer cells to taxane treatments using quartz crystal microbalance biosensor technology. Assay Drug Dev. Technol..

[29] Danowski B.A. (1989). Fibroblast contractility and actin organization are stimulated by microtubule inhibitors. J. Cell Sci..

[30] Kolodney M.S., Elson E.L. (1995). Contraction due to microtubule disruption is associated with increased phosphorylation of myosin regulatory light chain. Proc. Natl. Acad. Sci. USA.

[31] Ingber D.E. (1993). Cellular tensegrity: Defining new rules of biological design that govern the cytoskeleton. J. Cell Sci..

[32] Stamenović D., Mijailovich S.M., Tolić-Nørrelykke I.M., Chen J., Wang N. (2002). Cell prestress. II. Contribution of microtubules. Am. J. Physiol. Cell Physiol..

[33] Su X., Zhang L., Kang H., Zhang B., Bao G., Wang J. (2019). Mechanical, nanomorphological and biological reconstruction of early-stage apoptosis in HeLa cells induced by cytochalasin B. Oncol. Rep..

[34] Pastrana H.F., Cartagena-Rivera A.X., Raman A., Ávila A. (2019). Evaluation of the elastic Young’s modulus and cytotoxicity variations in fibroblasts exposed to carbon-based nanomaterials. J. Nanobiotechnol..

[35] Cai X., Xing X., Cai J., Chen Q., Wu S., Huang F. (2010). Connection between biomechanics and cytoskeleton structure of lymphocyte and Jurkat cells: An AFM study. Micron.

[36] Riveline D., Zamir E., Balaban N.Q., Schwarz U.S., Bershadsky A.D. (2001). Focal contacts as mechanosensors. J. Cell Biol..

[37] Carisey A., Tsang R., Greiner A.M., Nijenhuis N., Heath N., Nazgiewicz A., Kemkemer R., Derby B., Spatz J., Ballestrem C. (2013). Vinculin regulates the recruitment and release of core focal adhesion proteins in a force-dependent manner. Curr. Biol..

[38] Goldmann W.H., Ezzell R.M. (1996). Viscoelasticity in wild-type and vinculin-deficient (5.51) mouse F9 embryonic carcinoma cells examined by atomic force microscopy and rheology. Exp. Cell Res..

[39] Samuels M., Ezzell R.M., Cardozo T.J., Critchley D.R., Coll J.L., Adamson E.D. (1993). Expression of chicken vinculin complements the adhesion-defective phenotype of a mutant mouse F9 embryonal carcinoma cell. J. Cell Biol..

[40] Bertheloot D., Latz E., Franklin B.S. (2021). Necroptosis, pyroptosis and apoptosis: An intricate game of cell death. Cell. Mol. Immunol..

[41] Poreba M., Strózyk A., Salvesen G.S., Drag M. (2013). Caspase substrates and inhibitors. Cold Spring Harb. Perspect. Biol..

[42] Oropesa-Ávila M., de la Cruz-Ojeda P., Porcuna J., Villanueva-Paz M., Fernández-Vega A., de la Mata M., de Lavera I., Rivero J.M.S., Luzón–Hidalgo R., Álvarez-Córdoba M. (2017). Two coffins and a funeral: Early or late caspase activation determines two types of apoptosis induced by dna damaging agents. Apoptosis.

[43] Qiu S., Hu Y., Dong S. (2021). Pan-cancer analysis reveals the expression, genetic alteration and prognosis of pyroptosis key gene GSDMD. Int. Immunopharmacol..

