# Supplementary material for: A Novel Method in Identifying Pyroptosis and Apoptosis Based on the Double Resonator Piezoelectric Cytometry Technology
Source: Biosensors (Basel). 2023 Mar 7;13(3):356. doi: 10.3390/bios13030356 (PMC10046136; doi:10.3390/bios13030356)
Supplement: Supplementary file 1 [file biosensors-13-00356-s001.zip › biosensors-2230846-supplementary.pdf]

biosensors

MDPI

Supporting information

A Novel Method in Identifying Pyroptosis and Apoptosis Based on the Double Resonator Piezoelectric Cytometry Technology

Wenwei Li, Jing Li, Yanyang Wu and Tiean Zhou

Table S1. Methods for detecting both cell apoptosis and pyroptosis.

Technique	Indicators	Real-time	Non-invasive	Quantitative
Microscopy analysis/Scanning electron microscopy/Transmission electron microscopy	Cell morphology changes		√	
MTT/MTS assay	Cell viability			√
Flow cytometry/Fluorescence Microscopy	Staining status			√
WesternBlotting/ Immunofluorescence/Q-PCR	Molecular Biomarkers			
TUNEL method	DNA fragmentation			√
DRPC technology	Cell mechanical changes	√	√	√

Table S2. Mean values of mechanical parameters during HeLa pyroptosis and apoptosis.

Cells condition		Time Region (h)	ΔS (dyne/cm)	G′ (pascal)	G′′ (pascal)
LPS + CTB	Cell adhesion stage	10.0 ~ 14.5	14,378.9±1822.4	3684.1±125.1	45,161.6±185.4
	Pyroptosis stage	24.0 ~ 27.0	- 8850.1±5965.5	17,326.1±1616	60,754.8±1757.1
TNF + CHX	Cell adhesion stage	10.0 ~ 12.5	13,333.1±1683.3	9165.8±81.8	52,352.8±247.9
	Apoptosis stage	24.0 ~ 27.0	20,865.9±3999.0	2784.8±710.8	47,958.0±1354.4

Table S3. Mean values of mechanical parameters during GSDMD$^{-/-}$ -DEVD- HeLa non-pyroptosis and pyroptosis.

Cells condition		Time Region (h)	ΔS (dyne/cm)	G′ (pascal)	G″ (pascal)
LPS + CTB	Cell adhesion stage	10.0 ~ 14.5	6324.1±943.5	52,127.5±1122.3	83,604.9±921.6
	Non-Pyroptosis stage	24.0 ~ 27.0	21,173.9±1637.6	60,754.8±1757.1	108,792.2±421.0
TNF + CHX	Cell adhesion stage	10.0 ~ 12.5	13,861.7±1193	1813,5±147.8	42,390.7±678.9
	Pyroptosis stage	24.0 ~ 27.0	-19,248.3±4764.8	1891.9±304.4	44,258.3±306.5
